# Whole-Genome Sequence Analysis and Subtractive Screening of Lactobacilli in the Searching for New Probiotics to Protect the Mammary Glands

**DOI:** 10.3390/ijms262110809

**Published:** 2025-11-06

**Authors:** Dobroslava Bujňáková, Tímea Galambošiová, Lívia Karahutová

**Affiliations:** Institute of Animal Physiology, Centre of Biosciences of the Slovak Academy of Sciences, Šoltésovej 4/6, 040 01 Košice, Slovakia; dbujnak@saske.sk (D.B.); kocurekova@saske.sk (T.G.)

**Keywords:** *Lactobacilli*, MALDI-ToF MS, enzyme activity, antibiotic resistance, antimicrobial activity, biofilm, WGS

## Abstract

To discover new probiotics that can protect mammary glands from mastitis, 40 *Lactobacillus* (*Ligilactobacillus*) spp. isolates from bovine milk were subjected to a preliminary series of in vitro subtractive analyses. Antibiotic susceptibility testing was performed according to the ISO norm 10932. Many lactobacilli had elevated MIC values for kanamycin (35%), but fewer were resistant to chloramphenicol (15%), streptomycin (7.5%) and tetracycline (5%). The enzymic activities of lactobacilli were tested using an API ZYM system. Nearly 27% exhibited undesirable activities (β-glucuronidase, β-glucosidase and N-acetyl-β-glucosaminidase). The safe strains were monitored for antimicrobial activity against *Staphylococcus aureus*, *Escherichia coli*, *Salmonella enteritidis*, and *Bacillus cereus* using microtiter plates and for their ability to form biofilms using the crystal violet assay. The antimicrobial activity of lactobacilli against indicator bacteria ranged from 29 to 89% and the isolates exhibited moderate-to-high biofilm formation. Suitable strains were selected for whole-genome sequencing analysis. Antibiotic-resistance genes and putative virulence genes were not predicted in the genomic analysis. Moreover, the isolate *Ligilactobacillus salivarius 48* carries genetic information responsible for bacteriocin production that is similar to that encoding salivaricin CRL1328. Our study demonstrates the safety of the above mentioned isolate, which has potential to be used as a probiotic, exerting health benefits through production of antimicrobial substances.

## 1. Introduction

Mammary gland disease, also known as bovine mastitis (BM), is defined as an inflammatory disease of the mammary gland; it is most often caused by pathogenic bacteria. BM can arise from inflammation of the mammary gland and milk ducts, incorrect technological procedures during milking, metabolic disorders, or udder injuries [1]. The causative agents of BM can be divided into two major groups: the first includes contagious pathogens (e.g., *Staphylococcus aureus* and *Streptococcus agalactiae*) and the second environmental pathogens (e.g., *Escherichia coli,* coagulase-negative *staphylococci*, and *Pseudomonas* spp.) [2,3].

BM leads to changes in the composition and quality of milk, as well as reduced milk yields [4]. Moreover, it is recognized as the most widespread disease in the dairy industry, contributing to significant economic losses [5]. In practice, BM is commonly treated via intramammary antibiotic therapies, systemic administration (parenteral) of antimicrobial agents, or a combination of both strategies to increase therapeutic efficacy [3]. Emerging problems in the treatment of BM include the proliferation of antimicrobial-resistant pathogenic bacteria, resulting in frequent recurrence of intramammary infections, and permanent damage to mammary gland tissue, which can compromise udder health and lead to significant reduction in milk yield. In addition, incorrect withdrawal periods or the use of antimicrobials outside of approved indications can lead to harmful residues in milk, posing potential risks to public health and compromising the quality of milk intended for human consumption [6]. The growing problem of antimicrobial resistance in the treatment of BM has stimulated interest in alternative therapeutic and preventive approaches. These include antimicrobial peptides, bacteriophages, non-steroidal anti-inflammatory drugs, phytotherapeutics, and probiotics, which have shown potential for modulating the host immune response, promoting udder health, and improving regeneration [7].

Probiotics are live microorganisms that, when administered in adequate quantities, confer a health benefit on the host [8]. Most of the common probiotic strains are from *Lactobacillus*, *Lactiplantibacillus*, *Enterococcus*, and *Saccharomyces* genera. For the prevention of BM, the study of probiotic lactobacilli strains has emerged as a significant research area based on their ability to combat mastitis. Lactobacilli can protect the mammary gland through different mechanisms, including antimicrobial activity through the production of compounds such as bacteriocins (ribosomally synthesized antimicrobial peptides) or metabolites (lactic acid, hydrogen peroxide) and modulation of the host’s immune response. Recent evidence demonstrates that some probiotic strains can modulate Toll-like receptor (TLR)-driven inflammation in the mammary gland [9].

The ideal probiotic lactobacilli for use against BM must meet several key requirements, encompassing specific characteristics of the bacterial strain, safety, and efficacy [10]. The suitable standard for characterizing potential probiotic lactobacilli strains is a combination of classical screening methods and whole genome sequencing (WGS). Using classical screening as a first step can help to exclude many strains with inappropriate properties (such as unsuitable enzymic activity, antibiotic resistance, or a low antimicrobial effect). The advantage of using classical lab methods to study antimicrobial activity or ability to form biofilms (adhesion) in vitro directly confirms or refutes the physical/measurable/visible capabilities of specific strains. This constitutes an advantage over WGS, which can only predict these properties. On the other hand, WGS provides a complete set of genes related to virulence factors and transferable antibiotic resistance, allowing assessment of the safety of tested strains. This method helps minimize the likelihood using unsafe strains. Therefore, this study employed a subtractive in vitro screening system to evaluate the safety and functional probiotic properties of selected bacterial isolates. The analysis focused on key characteristics, including antibiotic susceptibility profiles, enzymatic activity, and antagonistic potential against selected BM-associated pathogens, with the aim of identifying promising candidates for future application in bovine udder health management. Following the exclusion of strains exhibiting undesirable traits or limited antimicrobial activity, the selected isolate *Ligilactobacillus salivarius* 48 was subjected to WGS analysis.

## 2. Results and Discussion

### 2.1. Bacterial Identification

In accordance with microbiology methodologies, 130 bovine milk samples were examined for presence of lactobacilli. One or two of typical colonies from Rogosa agar (small- to medium-sized, circular, raised, and with a greyish-white color) for each milk sample were identified via Matrix-Assisted Laser Desorption Ionization–Time of Flight Mass Spectrometry (MALDI-ToF MS). A total of 103 isolates were successfully identified with a score > 2. *Ligilactobacillus salivarius* was the most abundant (22 isolates), followed by *Lactobacillus mucosae* (18 isolates), *Pediococcus* sp. (11 isolates), *Pediococcus acidilactici, Ligilactobacillus ruminis,* and *Lactobacillus fermentum* (*n* = 7), *Lactobacillus ingluviei* and *Lactobacillus paracasei* (*n* = 6), *Ligilactobacillus agilis* (*n* = 5), *Lactobacillus casei* and *Lactobacillus buchneri* (*n* = 3), and *Lactobacillus pontis* and *Lactobacillus paraplantarum* (*n* = 2). *Lactobacillus plantarum, Lactobacillus rhamnosus*, and *Ligilactobacillus acidipiscis* were represented by one isolate each. We also detected one isolate of *Bifidobacterium merycicum*. The remaining 27 isolates could not be identified. From these strains, a total of 40 lactobacilli were selected (on the basis of obtaining a MALDI-ToF MS score ≥ 2.3) for subtractive screening, including safety assessment and, more specific monitoring of phenotype antibiotic resistance patterns and bacterial enzyme activities.

### 2.2. Safety Assessment

#### 2.2.1. Phenotype Antibiotic Resistance

According to the European Food Safety Authority (EFSA) and the Panel on Additives and Products or Substances used in Animal Feed (FEEDAP), all microorganisms used as probiotics must have a specified sensitivity to reference antibiotics [11]. The minimum inhibitory concentrations [MICs; (μg/mL)] for reference antibiotics were interpreted in accordance with the EFSA [12]. A relatively large number of lactobacilli isolates (35%) had elevated MIC values for kanamycin [*Ligilactobacillus agilis* (3), *Ligilactobacillus salivarius* (12)] (MIC > 16 or 64 μg/mL on an individual level depending on species). Less frequently, the MIC values were higher for chloramphenicol (15%) (MIC > 4 or 8 μg/mL) in *Lactobacillus fermentum* (1), *Lactobacillus casei* (1), *Ligilactobacillus agilis* (1), *Lactobacillus ingluviei* (1), and *Ligilactobacillus salivarius* (2); and streptomycin (7.5%) (MIC > 16, 32, or 64 μg/mL) [*Ligilactobacillus agilis* (3)]. Sporadically, strains were resistant to tetracycline (5%) (MIC > 4, 8, 32 μg/mL). The strains were phenotypically sensitive to other antibiotics. Detailed results are provided in the Supplementary Material (Table S1). The distributions of the MIC, MIC_50_, and MIC_90_ values of eight antibiotics among the studied *Lactobacillus* (and *Ligilactobacillus)* spp. are presented in Table 1.

**Figure 1:**
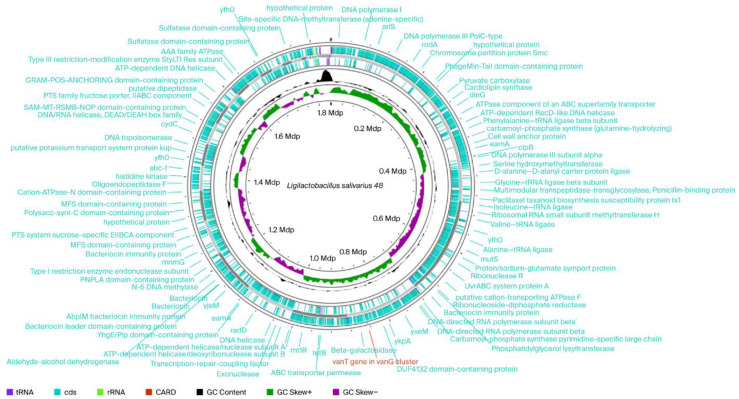
Circular chromosomal genome maps of *Ligilactobacillus salivarius* 48. Legends: From outside to centrum: Ring 1 represents CARD gene (1 match *van*T gen in *van*G cluster) and Rings 2 and 3 demonstrate the protein-coding genes (CDS) (blue), tRNA (plump purple), and rRNA (light green) on the forward and reverse strands, respectively; ring 4 represents the GC content plot; ring 5 represents the positive and negative GC skew in green and purple, respectively.

Most lactobacilli species are naturally resistant to aminoglycosides (gentamicin, kanamycin, streptomycin, and neomycin), ciprofloxacin, and trimethoprim and sensitive to penicillin and lactams, chloramphenicol, tetracycline, erythromycin, linezolid, and quinupristin-dalfopristin. However, some lactobacilli strains isolated from fermented foods have been shown to be resistant to tetracycline, erythromycin, clindamycin, and chloramphenicol [13]. Although phenotype of the strain cannot confirm the presence or absence of transferable resistance genes, some of the observed levels are compatible with common transferable determinants. This fact underscores the need to monitor the presence of transferable antibiotic resistance genes at the molecular level. For example, resistance to kanamycin and streptomycin is encoded by genes for nucleotidyl transferase enzymes, such as *ant*(6) and *ant*(9), or aminoglycoside-3′-phosphotransferase (APH(3′)), also known as aminoglycoside kinase. Generally, members of the *Lactobacillus* genus are sensitive to chloramphenicol [14]; however, some studies have reported MIC values above cut-off thresholds for this antibiotic [13,15]. Sequences associated with chloramphenicol resistance are encoded by chloramphenicol acetyltransferase, such as the *cat* gene, and specific membrane-associated transporters, such as CmlA. Tetracycline resistance is related to the presence of two gene groups. The first group is responsible for the production of membrane-associated proteins capable of mediating the antibiotic efflux through genes such as *tet*(Z), *tet*(K), *tet*(L), and *tcr*3. The second group of genes encode cytoplasmic proteins able to protect ribosomes from antibiotic attack [*tet*(M), *tet*(O), *te*t(S), *tet*(W), *tet*(Q), *tet*(T), and *otr*(A)] [16]. Vancomycin- a glycopeptide antibiotic—can inhibit cell wall synthesis in Gram-positive bacteria by binding to the D-alanyl-D-alanine precursor of the peptidoglycan, compromising the formation of cross-links [15]. Most lactobacilli possess endogenous enzymes capable of synthesizing D-lactate and binding it to peptidoglycan, thus inducing intrinsic resistance to vancomycin [14]. Although we did not monitor phenotypic resistance to vancomycin, whole- genome sequencing (WGS) analysis of *Ligilactobacillus salivarius* 48 revealed glycopeptide resistance gene clusters, e.g., the *vanT* gene in the *vanG* cluster (Figure 1), that, through The Comprehensive Antibiotic Resistance Database (CARD’s) Antibiotic Resistance Ontology, were identified as also being present in *Lactobacillus gasseri* (https://card.mcmaster.ca/ontology/39406 (accessed on 6 June 2025)) [17].

The recent publication of the genome sequences of almost all *Lactobacillus* species allows us to assess the safety of the *Lactobacillus* genus by monitoring the presence of antibiotic resistance genes and their potential for transfer to other microorganisms through strain sequencing. WGS can potentially identify all possible genetic determinants of antibiotic resistance in the microbial genome. Therefore, instead of monitoring genes using polymerase chain reaction, we subjected the selected *Lactobacillus salivarius* 48 to WGS.

#### 2.2.2. Enzyme Activities

All 40 lactobacilli isolates were assayed using API ZYM tests performed according to the manufacturer’s instructions. The results were graded from 0 to 5 by comparing the observed color with the color reaction chart. A value of 0–2 corresponded to a negative reaction or very low activity, whereas values of 3–5 indicate a positive reaction. The concentration of free hydrolyzed substrate can be estimated from the color intensity: 0–40 nmoL.

Of the 19 enzymatic activities investigated, 13 (alkaline phosphatase, esterase, leucine arylamidase, valine arylamidase, cystine arylamidase, acid phosphatase, phosphohydrolase, α-galactosidase, β-galactosidase, β-glucuronidase, α-glucosidase, β-glucosidase, β-glucosaminidase) were considered strong, i.e., with a score of 3–5. The other enzymatic activities were very low (10 nmoL or zero nmoL).

Of the lactobacilli strains examined, nearly 27.5% exhibited undesirable enzymatic activities, including β-glucuronidase, β-glucosidase, and N-acetyl-β-glucosaminidase activities, which are associated with harmful effects through the reactivation of carcinogens or alterations in drug disposition. For example, bacterial β-glucuronidase, can cleave glucuronic acid from conjugated compounds, such as those formed from carcinogenic chemicals, reinstating their toxicity and promoting reabsorption. Similarly, these bacterial enzymes play roles in altering drug and toxin metabolism in the gut [18]. We excluded strains exhibiting these activities from further research.

In contrast, β-galactosidase is a promising eligible enzyme that facilitates lactose digestion through catalyzing the hydrolysis of lactose into glucose and galactose [19]. Under specific conditions, β-galactosidase can also participate in the transglycosylation of lactose, producing prebiotics known as galactose-oligosaccharides, which are highly sought after [20]. The above mentioned enzymes isolated from lactobacilli have become popular among researchers and dairy industry stakeholders due to their high activity and high stability, the fact that they are food-grade standard, and because the microorganism that they are produced by can be grown easily in a time-efficient manner. The enzymatic activity patterns of our selected strains are shown in Table 2.

*Ligilactobacillus salivarius* 48 showed high β-galactosidase activity (40 nmoL) and, interestingly, based on WGS, was predicted to contain β-galactosidase hydrolase family genes involved in lactose hydrolysis (Figure 1).

#### 2.2.3. Biofilm Formation

Strains that form biofilms have higher probiotic potential due to the protective function of biofilm against to environmental conditions, pathogenic bacteria, and mechanical influence. Moreover, the therapeutic utilization of strains can more effective if the strain has the ability to form a biofilm [21]. The adhesion of strains on the surface of the udder can initiate an immune response, which is a desirable property of probiotics. The strains selected for our study had moderate-to-high biofilm formation. Absorbance values of over 0.3 were measure for the strains *Ligilactobacillus salivarius* 48 (0.375) and *Lactobacillus paracasei* 53 (0.369). *Ligilactobacillus salivarius* 100/3*, Lactobacillus fermentum* 106, *Ligilactobacillus salivarius* 105/2, and *Lactobacillus paracasei* 29 produced strong biofilms, with absorbance values of 0.294, 0.251, 0.223, and 0.217, respectively. Only one strain, *Lactobacillus paracasei* 1 exhibited a medium level of biofilm formation (0.128). Probiotic and pathogenic microorganisms complete for adherence sites on the surface of somatic cells [22]. Although bacterial adherence to cells was not investigated in our work, biofilm production can be an indicator of good bacterial adhesion.

#### 2.2.4. Antimicrobial Activity of Neutralized Cell Free Supernatant from Individual *Lactobacilli*

The antagonist effect of neutralized cell-free supernatants (NCFSs) obtained from seven lactobacilli was assessed against *Staphylococcus aureus* isolated from bovine mastitis and reference (indicator) strains (*Escherichia coli* C 1971, *Salmonella enteritidis* CCM 4420, and *Bacillus cereus* CCM 869). Table 3 summarizes the results, including mean (absorbance) ± SD (standard deviation) and statistical analysis (*t*-tests). Each indicator strain was incubated with the NCFSs of seven lactobacilli at a ratio of 1:1 (*v*/*v*).

Significant antimicrobial activity was observed against all indicator bacteria following 24 h of incubation with all NCFSs; the activity ranged from 29 to 89%. The NCFS from *Lactobacillus paracasei* 29 had relatively lower inhibitory activity (only 29% against *Bacillus cereus* CCM 869, and 56% against *Salmonella enteritidis* CCM 4420, and 57% against *Staphylococcus aureus*). *Escherichia coli* C 1971 was inhibited by all NCFSs by 66–68% (Table 3).

An additional method was used to confirm the viability of bacteria and the effect of NCFSs. After incubation in a 3×- concentrated Mueller Hinton (MH) broth (to ensure sufficient nutrients) containing NCFSs, we detected considerable inhibition of bacterial growth for all tested supernatants relative to control (without NCFS).

Numerous studies have demonstrated that cell-free supernatants (CFSs) from lactobacilli cultures demonstrate antimicrobial activity, primarily due to the presence of lactic acid, which causes a decrease in environmental pH, and bacteriocins, which are peptide-based toxins that target bacteria by disrupting their membranes [23]. The acid effect was removed by the neutralization of our CFSs. Nevertheless, the antimicrobial action remained significant; therefore, we assumed that these isolates produced antimicrobial peptides. All isolates were subjected to WGS. The partial results for *Ligilactobacillus salivarius* 48 are presented in the following section.

### 2.3. WGS of Ligilactobacillus Salivarius 48

The genome of *Ligilactobacillus salivarius* 48 has a total size of 1.841 Mbp. A total of 1 853 genes were predicted. Among them, 1750 genes were predicted to be protein-coding genes; the remaining 103 genes were transfer RNA (tRNA) (64 genes), ribosomal RNA (rRNA) (5 genes), transfer-messenger RNA (tmRNA) (1 gene), and other non-coding RNAs (31). A circular genome map was generated in the Proksee expert system (https://proksee.ca/, accessed on 12 August 2025) for genome assembly, annotation, and visualization (Figure 1).

Based on the NCBI genome database (https://www.ncbi.nlm.nih.gov/genome, accessed on 11 May 2023, United States), 22 complete genome maps of *Ligilactobacillus salivarius* have been constructed [21]. For example, Yang et al. [24] determined the whole genome of *Ligilactobacillus salivarius* AR809; it contains 1967 genes, of which a total of 1593 genes encode proteins, whereas there are 79 tRNA genes and 22 rRNA genes on the circular chromosome and 240, 28, 3, and 2 protein-encoding genes were found on plasmids pA-pD, respectively.

The Average Nucleotide Identity (ANI) and digital DNA-DNA hybridization (DDH) values of genome sequences from *Ligilactobacillus salivarius* 48 and 15 other *Ligilactobacillus salivarius* isolates were determined, and the results are listed in the Supplementary Material (Table S2). ANI data indicate the similarity between two or more genomes at the nucleotide level. This metric is used primarily to verify and classify bacterial species; a threshold of 95% ANI is commonly used to define the same species. All our data fulfill this criterion, which, together with the fact that DDH values were above 70%, highlights the high degree of similarity between different *Ligilactobacillus salivarius* strains.

**Figure 2:**
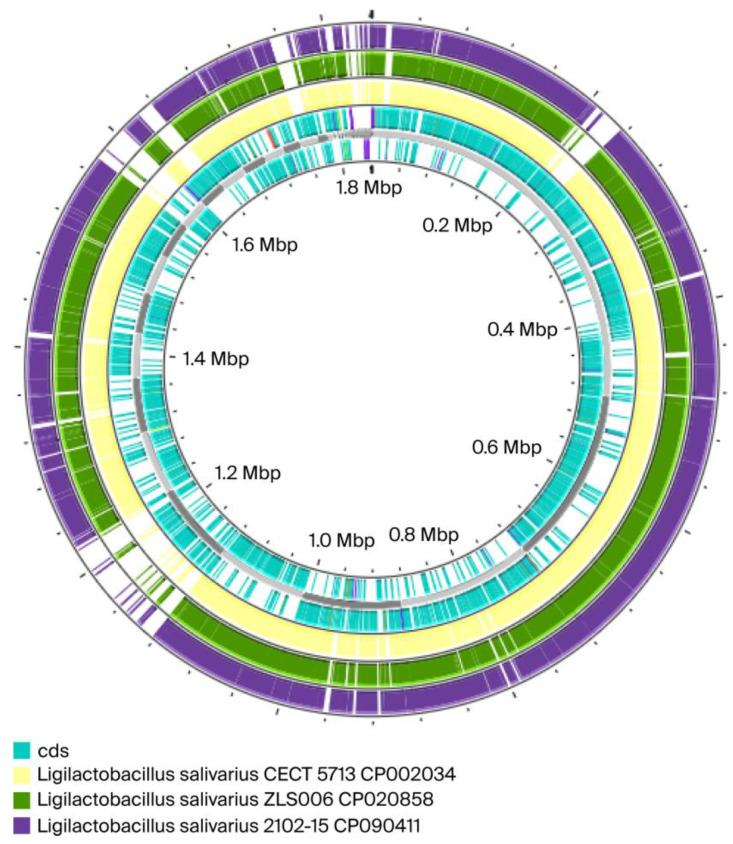
Comparisons between the genome of our isolate *Ligilactobacillus salivarius* 48 (in blue) and three others *Ligilactobacillus salivarius*: in yellow *Ligilactobacillus salivarius* CECT 5713 CP002034; in green *Ligilactobacillus salivarius ZLS006 CP020858*; in dark purple *Ligilactobacillus salivarius 2102-15 CP090411*. Our isolate 48 is represented by the cds (Coding Sequence).

The Proksee expert system was used to compare genome structures. Proksee uses a combination of analysis tools and an interactive, high-performance genome browser to perform genome structure comparisons. Users can upload their own data or use a GenBank accession to generate a graphical map, then perform comparisons by adding data from embedded analysis tools, such as BLAST, to the map. The system also allows for direct sequence searching and comparison with other genomes, making it possible to identify similarities and differences (Figure 2).

The results from ANI and DDH analyses and the genome map confirmed that *Ligilactobacillus salivarius* 48 is similar to *Ligilactobacillus salivarius* CECT 571 (ANI = 97.40%; DDH = 74.44%). These values indicated that the strains were same species but possibly different subspecies, as there are gaps in the map from the partially incomplete genomes of the isolates. The highest ANI and DDH values compared to our isolate were for *Ligilactobacillus salivarius* DSM 20555 = ATCC 11741 DSM 20555 [25] (ANI = 97.95%; DDH = 80.92%) (see Supplementary Material—Table S2). ANI values of *>*95% and DDH values of *>*70% are considered to indicate the same species [26].

ResFinder, which is used for the identification of acquired genes and/or finding chromosomal mutations facilitating antimicrobial resistance in microbial DNA sequences, did not predict any threats in *Ligilactobacillus salivarius* 48. Clustered Regularly Interspaced Short Palindromic Repeats (CRISPRs) and CRISPR-associated protein (Cas), which protect against mobile genetic elements (MGEs), were not detected in the mentioned strain. Plasmid sequences searching and mobilome prediction through PlasmidFinder revealed no matching records. Putative virulence factors were not found by genome sequencing (Tables S3–S7).

**Figure 3:**
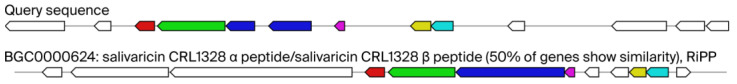
Query sequence of our isolate *Ligilactobacillus salivarius* 48 and their similarity with known cluster blast for salivaricin CRL1328 production gene clusters. Legends: AntiSMASH predicted biosynthetic gene clusters; known cluster blast salivaricin CRL1328 identified in *Ligilactobacillus salivarius* CRL 1328 isolated from the human vagina as 50% similar gene cluster against our query sequence. Genes with high homology are color-coded identically.

The antiSMASH bacterial program (version 8.0.0) identified secondary metabolite regions when using specific settings (e.g., strictness ‘relaxed’) and revealed significant hits for BGC0000624 salivaricin CRL1328 α peptide/salivaricin CRL1328 β peptide [27] (in detail: type ribosomal RiPP protein; number of proteins with BLAST (Basic Local Alignment Search Tool, version 2.17.0) hits to this cluster: 7; cumulative BLAST score 1390.0; the best Blast hit with ABQ84445.1: salivaricin induction peptide with 100% identity,100% coverage, and e-value 2.84 × 10^−24^) (Figure 3; Table S8).

#### 2.3.1. Prediction of the Salivaricin Gene Cluster

Figure 3 shows our query sequence for *Ligilactobacillus salivarius* 48 and its similarity with bacteriocin—salivaricin CRL1328 production gene clusters identified in human vaginal *Ligilactobacillus salivarius* CRL 1328. As you can see 50% of genes show similarity. In total seven proteins were predicted in region 1 of *Ligilactobacillus salivarius* 48 [bacteriocin-like prepeptide (start at 313 and end at 570); salivaricin CRL 1328 α-peptide (576–770); salivaricin induction peptide (1424–1540); sensory transduction histidine kinase (1540–2829); salivaricin response regulator (2843–3637); and hypothetical membrane protein (4064–6223)]. For detailed information pertaining to this, see Table S8.

**Figure 4:**
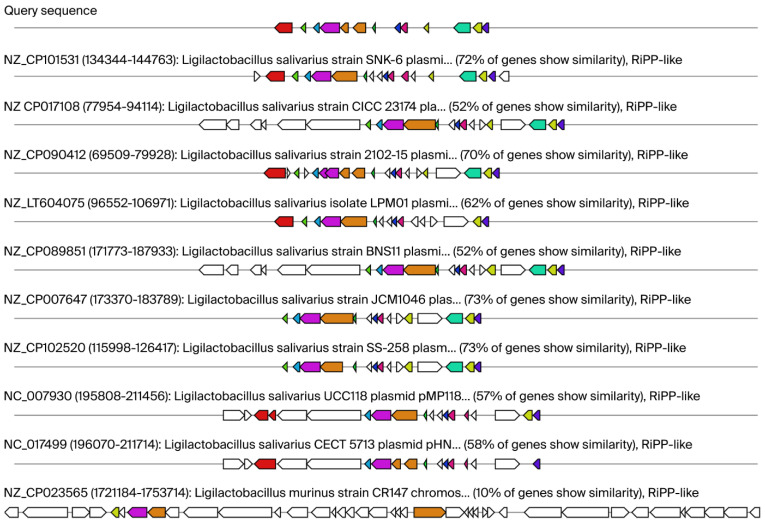
Putative regions containing gene clusters associated with ribosomally synthesized and post-translationally modified peptides (RiPP)- like proteins. Legends: The alignment, conducted via Cluster-BLAST (Basic Local Alignment Search Tool), revealed gene clusters that are related to the queried gene cluster. Specifically, the top ten matches to the RiPP- like proteins gene cluster from the various *Ligilactobacillus salivarius* and *Ligilactobacillus murinus* strains are shown. Genes with high homology are color-coded identically.

Figure 4 illustrates the alignment, conducted using Cluster-BLAST (Basic Local Alignment Search Tool, version 2.17.0). The figure shows the top ten matches to the RiPP- like protein gene cluster from the various *Ligilactobacillus salivarius* and *Ligilactobacillus murinus* strains in comparison with our query sequence.

**Figure 5:**
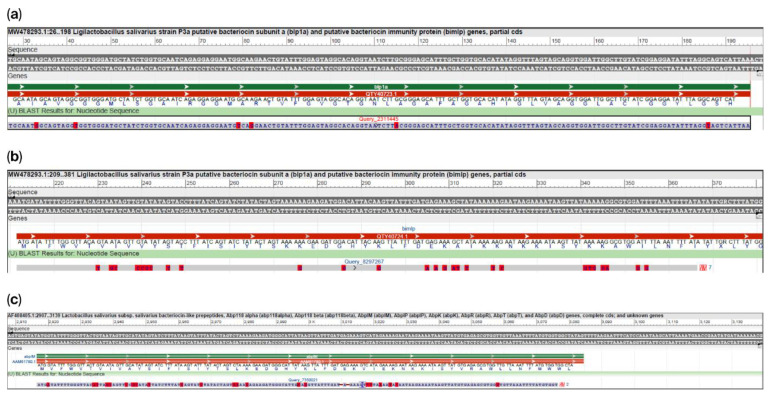
Sequence alignment of genes predicted for three bacteriocins with their corresponding full-length genes. (**a**): the 198 bp coding sequence for Blp1a has 97% identity to our query; coverage 80%; 6 mismatches (red highlight letters) compare to *Ligilactobacillus salivarius* strain P3a putative bacteriocin subunit a. (**b**): the 172 bp coding sequence for Bimlp with 83% identity to our query; 96% coverage; gaps 0; mismatches 27 (red highlight letters) compare to *Ligilactobacillus salivarius* strain P3a putative bacteriocin immunity protein. (**c**): the 232 bp coding sequence for AbpIM has 86% identity to our query; coverage 98%; 3 gaps; 2 unaligned regions; 22 mismatches (red highlight letters) compare to *Ligilactobacillus salivarius* subspecies salivarius bacteriocin-like prepeptide AbpIM.

The Proksee expert system revealed a specific 6 cds region of DNA that contains the instructions for building bacteriocins, including bacteriocin immunity protein; AbpIM bacteriocin immunity protein; bacteriocin leader domain-containing protein; and putative bacteriocin subunit a or Blp family class II bacteriocin. The specific regions obtained were compared using Blast (https://blast.ncbi.nlm.nih.gov/Blast.cgi (accessed on 20 October 2025) to acquire sequences producing significant alignments. We found three similar sequences: a 232 bp coding sequence for AbpIM (Abp118 immunity protein) with 86% identity to our query compared to the *Ligilactobacillus salivarius* subspecies salivarius bacteriocin-like prepeptide AbpIM; a 198 bp coding sequence for Blp1a (putative bacteriocin subunit a) with 97% identity to our query compared to the *Ligilactobacillus salivarius* strain P3a putative bacteriocin subunit; and a putative bacteriocin immunity protein (Bimlp) with 83% identity to our query compared to the *Ligilactobacillus salivarius* strain P3a putative bacteriocin immunity protein (Figure 5).

Generally, *Ligilactobacillus salivarius* strains possess antagonistic properties against various bacterial pathogens. This ability is attributed to the production of lactic acid, H_2_O_2_, and bacteriocins, as well as the capacity to colonize the gut for extended periods, leading to the exclusion of unfavorable microflora [28]. *Ligilactobacillus salivarius* 48 was predicted to carry genetic information similar to that encoding salivaricin CRL1328. For these two-component antibiotics, the α and β units work together to permeabilize the cell membrane of the target cell. The probable mode of action of salivaricin appears to be through pore formation, as evidenced by experiments with salivaricin mmaye1 [29].

A BLAST comparison of the specific DNA region obtained from the Proksee expert system, containing information for predicted bacteriocins, revealed high similarity with AbpIM (Abp118 immunity protein), Blp1a (putative bacteriocin subunit a), and putative bacteriocin immunity protein (Bimlp).

The Abp-118 immunity protein is encoded by the *abp*IM gene. This gene is located near the gene for the bacteriocin Abp-118 and provides protection to *Lactobacillus salivarius* from its own bacteriocin. Abp-118, a small heat-stable bacteriocin, is a class IIb two-peptide bacteriocin composed of Abp118α, which exhibits the antimicrobial activity, and Abp118β, which enhances the antimicrobial potential. Abp118 is an antimicrobial peptide with broad-spectrum activity against bacteria, including *Bacillus*, *Listeria*, *Enterococcus* and *Staphylococcus* [30].

Blp1a is a Blp (Bacteriocin-like peptide) family class II peptide. These bacteriocins are often produced by bacteria like Streptococcus. *Streptococcus salivarius* typically harbors a main chromosomal *blp* locus completed by several secondary loci that encode class II salivaricins with double-glycine maturation sites. They show only limited similarity with several class IIb two-component bacteriocins [31].

The bimlp gene encodes a bacteriocin-immunity protein, not the bacteriocin itself. This bacteriocin-immunity protein is one component of a broader gene cluster that also comprises bacteriocin-like genes *(blp*1a and *blp*1b) and likely helps the bacterium to be protected from its own bacteriocin, enabling it to remain viable while damaging other bacteria.

In the human oral strain *Lactobacillus salivarius* BGHO1, Busarcevic et al. [32] found a novel member of the class IId bacteriocins that was similar to putative bacteriocins from several oral streptococci. The high similarity of this bacteriocin with putative bacteriocins derived from several recent genomic projects of oral streptococci can be explained by horizontal gene transfer between species that share the same habitat.

#### 2.3.2. Prediction of the Bacterial Type III Polyketide Synthases Gene Cluster

**Figure 6:**
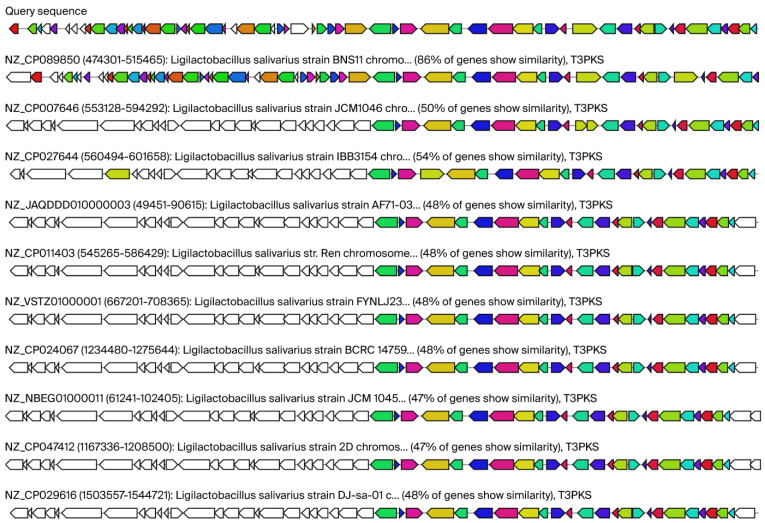
Putative regions containing gene clusters associated with bacterial type III polyketide synthases (T3PKS). Legends: The alignment, conducted via Cluster-BLAST (Basic Local Alignment Search Tool), revealed gene clusters that are related to the queried gene cluster. Specifically, the top ten matches to the T3PKS- like proteins gene cluster from the various *Ligilactobacillus salivarius* strains are shown. Genes with high homology are color-coded identically.

AntiSMASH analysis also predicted so-called bacterial type III polyketide synthases (T3PKSs), which are involved in the biosynthesis of some lipidic compounds and various secondary metabolites. Many compounds produced by bacterial T3PKSs have significant biological functions. They can act as signaling molecules and have potential pharmaceutical properties, such as antimicrobial or anticancer activities [33]. The low similarity of our *Ligilactobacillus salivarius* 48 query to known T3PKSs clusters could mean we have uncovered novel bioactive molecules with therapeutic potential. Figure 6 illustrates putative regions containing gene clusters associated with T3PKS (the top ten matches to the T3PKS- like protein gene cluster from the various *Ligilactobacillus salivarius* strains to our query sequence are shown).

## 3. Materials and Methods

### 3.1. Bacterial Isolation and Identification

A total of 130 bovine milk samples collected from cows without a clinical manifestation of BM were examined for the presence of lactobacilli strains. The individual milk samples (100 µL) were plated onto Rogosa agar (Oxoid, UK) under anaerobic conditions and incubated at 37 °C for 48 h. One or two of typical colonies (small-to medium-sized, circular, raised, and with a greyish-white color) from each milk sample were picked, analyzed and assessed using MALDI-ToF MS (Bruker Daltonik GmbH, Leipzig, Germany), as described by Bessède et al. [34]. To identify microorganisms, the raw spectra data obtained for each isolate were imported into BioTyper software, version 2.0 (Bruker Daltonic). Next, only *Lactobacillus* (or *Ligilactobacillus*) sp. strains with a MALDI-ToF MS score ≥ 2.3 were selected. Isolated lactobacilli strains were stored in sterile cryotubes (KRYOBANKA B, ITEST plus s.r.o., Hradec Králové, Czech Republic) at –80 °C until further analysis.

### 3.2. Antibiotic Susceptibility Testing

The MICs of selected antibiotics were determined using the microdilution method according to the international standard (ISO 10932/IDF 223) [35]. More specifically, a VetMIC Lact 1 plate (Statens Veterinarmedicinska Anstalt, Uppsala, Sweden) for lactic acid bacteria, containing serial 2-fold dilutions of 8 antibiotics was used. The MIC ranges for specific antibiotics varied and fell within the concentration ranges of the antibiotics tested: for gentamicin 0.5–256 μg/mL; for kanamycin 2–1024 μg/mL; for streptomycin 0.5–256 μg/mL; for tetracycline 0.125–64 μg/mL; for chloramphenicol 0.125–64 μg/mL; for neomycin 0.5–256 μg/mL; for erythromycin 0.016–8 μg/mL; and for clindamycin 0.032–16 μg/mL. VetMIC Lact 1 plate were incubated for 48 h at 28 °C in anaerobic conditions. The interpretation of results was conducted based on visual assessment and a comparison of every well with positive control of plates. Based on these values, MICs were determined. The MICs were evaluated according to the specific criteria described in EFSA guidelines [12].

### 3.3. Enzyme Activities

The enzyme activities of 19 hydrolytic enzymes (alkaline and acid phosphatases, esterase, esterase lipase, lipase, leucine, valine and cystine arylamidases, trypsin, α-chymotrypsin, phosphohydrolase, α-galactosidase, β-galactosidase, β-glucuronidase, α-glucosidase, β-glucosidase, β-glucosaminidase, α-mannosidase, and α-fucosidase) were analyzed using the special microenzyme API ZYM system (BioMérieux, Marcy-l’Étoile, France). The method described by Arora et al. [36] was used to ensure the correct enzymatic reactions. Samples were incubated at 37 °C for 4h. Subsequently, Zym A reagent was added to terminate the reaction and Zym B reagent was added to produce the color. The results were characterized after 5 min by comparing the colors with the color scale included in the kit. Color scores (0–5) and their corresponding approximate nanomolar values were determined based on the manufacturer’s instructions.

### 3.4. Biofilm Production

Individual colonies from the strains were inoculated to 5 mL of Rogosa broth (Oxoid, UK) and incubated at 37 °C for 24h. Subsequently, tubes with bacteria were centrifugated for 10 min at 10 000 rpm. The 1 McFarland suspension (corresponding to 1.5–3 × 10^8^ CFU/mL) was created by resuspending the bacterial sediment in Phosphate-Buffered Saline (PBS; Oxoid, Basingstoke, Hampshire, UK). Microtitrate plates were used to assess the production of biofilms (Nunc, Roskilde, Denmark); 100 µL of bacterial suspension was added to every well before incubation for 48 h at 37 °C. The coloring of plates was performed using a modified method to that described by Bujňáková et al. [37]. The first step was washing the plates multiple times with 200 µL of PBS, followed drying them upside down at 37 °C for 30 min. After drying, a 1% solution of crystal violet (Mikrochem, Pezinok, Slovakia) in ethanol was added to the wells incubating for a further 15 min at 25 °C. The next step was aspirating the dye, washing multiple times with distilled water, and drying at 25 °C for 10 min. A 200 µL volume of ethanol-acetone (80:20) solution was added to the wells. Absorbance measurement was performed at wavelength λ = 570 nm (A_570_) using the Synergy HT Multi-Mode Microplate Reader (BioTek, Winooski, VT, USA). Ethanol-acetone solution was used as a blank. Biofilm production capacity was categorized into three groups: low (A_570_ < 0.1), moderate (A_570_ = 0.1–0.2) and high (A_570_ > 0.2). Data were obtained from independent experiments with at least eight replicates and expressed as mean A_570_ ± SD (standard deviation). More detailed information is provided in the Statistical Analysis section.

### 3.5. Antimicrobial Activity of NCFSs from Individual Lactobacilli

The antimicrobial activity of CFSs from lactobacilli was tested on *Staphylococcus aureus* isolated from bovine mastitis and selected reference strains (*Escherichia coli* C 1971, *Salmonella enteritidis* CCM 4420, and *Bacillus cereus* CCM 869) that were kindly provided by the University of Veterinary Medicine and Pharmacy in Košice.

To obtain CFSs, lactobacilli colonies (cultured on Rogosa agar) were inoculated in De Man–Rogosa–Sharpe (MRS) broth (Oxoid, UK) and incubated for 48 h at 37 °C. After incubation, individual MRS broth samples were centrifuged at 4000 rpm for 10 min, before CFSs were harvested, filtered using 0.22 µM cellulose acetate membranes, alkalinized by adding 1 M NaOH (reaching pH = 7) to obtain NCFSs, and then stored at −80 °C until use. An NCFS aliquot was plated onto MRS agar (Oxoid, UK) and cultured anaerobically for 48 h at 37 °C to confirm it was free of lactobacilli.

The antagonism experiment was performed in a sterile 96-well microtiter plate (Nunc, Roskilde, Denmark). Each well contained 100 µL of indicator (reference) strains at 1.5–3 × 10^8^ CFU/mL in PBS, with 100 µL of sterile Mueller Hinton (MH) broth and 100 µL of NCFSs from individual lactobacilli. The microtiter plate was incubated at 37 °C under aerobic conditions and measured after 24 h. The absorbance at wavelength λ = 600 nm (A_600_) of the medium in each well was evaluated using a spectrophotometer Synergy HT Multi-Mode Microplate Reader (BioTek, Winooski, VT, USA). Indicator (reference) strains without NCFS were used as controls. Data were generated from independent experiments with at least eight replicates and expressed as mean A_600_ ± SD. More detailed information is provided in the Statistical Analysis section.

In addition, the NCFS effects were tested using another method to confirm the viability of tested bacteria. Concentrated MH broth (3×) was prepared to ensure that any observed inhibition was due to antimicrobial activity and not a deficiency of nutrients. The experiment was carried out in a total volume of 0.9 mL, containing from 600 µL of the individual NCFSs (2 parts) and 300 µL of 3× MH broth (1 part). Bacteria were inoculated into the solution at 1.5% of the total volume. As a positive control, 600 µL of sterile MRS broth and 300 µL of 3×MH with bacteria (as mentioned above) was used. The test tubes were incubated at 37 °C. Bacterial growth/viability after 24 h was assessed using the agar plate method. A 100 µL volume was streaked onto MH agar plates, which were then incubated at 37 °C for 24 h.

### 3.6. Extraction of Genomic DNA

Isolation of genomic DNA from lactobacilli with no undesirable traits and strong antimicrobial ability (seven isolates) was performed using the DNeasy PowerLyzer Microbial Kit (Qiagen GmbH, Hilden, Germany). Firstly, bacterial cultures were resuspended in an appropriate solution and then added to a beating tube. Next, bacteria were lysed via a combination of heat and detergent in lysis solution. The released DNA was entrapped on a silica gel membrane in a centrifugal column, then washed and eluted according to kit instructions. The obtained DNA was subjected to quantity and quality control using a NanoDrop 2000 c Spectrophotometer (ThermoFisher Scientific, Wilmington, MA, USA) based on the absorbance value A_260_ (1.0) = 50 μg/mL (for quantity) and ratio A_260/A280_ = 1.8–2.0 (for quality). All isolated DNA met the WGS criteria or quantity and quality.

### 3.7. WGS

A sequencing library for the Illumina platform was prepared using the QIAseq FX DNA Library UDI Kit (QIAGEN, Hilden, Germany). Sequencing was performed on a NovaSeq X Plus system (Illumina, San Diego, CA, USA) using short-read technology. Raw reads were trimmed using Trimmomatic v0.39 to remove adaptor residues and discard low-quality read regions (Q ≤ 20) [38]. The resulting high-quality trimmed reads were assembled de novo using SPAdes v3.11.0 [39] to generate contigs in FASTA format.

### 3.8. WGS Characterization

After the de novo assembly process was completed, WGS data were characterized using the NCBI prokaryotic genome analysis channel to predict protein-coding genes and genes for non-coding RNAs, such as rRNA and tRNA [40].

ANI and DDH values were calculated and compared using the JSpeciesWS web server tool (Version: 3.8.4) [41] and Genome-Genome Distance Calculator (GGDC version 2.1) BLAST [42]. Additionally, a circular map was constructed using the Proksee expert system to assess homology between our isolate and 3 other *Ligilactobacillus salivarius* strains*: CECT 5713 CP002034, ZLS006 CP020858*, and *2102-15 CP090411*.

Additionally, various bioinformatic tools were used for the WGS data characterization. For the prediction of antimicrobial resistance genes (ARGs), WGS data (in FASTA format) were analyzed under default settings (threshold for 95% identity, 60% minimum length with selected species, and others) in the web server ResFinder version 4.7.2 [43]. ResFinder identifies acquired genes and/or finds chromosomal mutations facilitating antimicrobial resistance in total or partial bacterial DNA sequences.

CRISPR-Cas system (Clustered Regularly Interspaced Short Palindromic Repeats- CRISPR-associated proteins) was investigated using CRISPRCasFinder (https://crisprcas.i2bc.paris-saclay.fr/CrisprCasMeta/Index, version 4.2.17 (accessed on 24 June 2025)). CRISPRCasFinder enables easy detection of CRISPR and Cas genes in user-submitted sequence data.

Plasmid sequences were detected using PlasmidFinder software, version 2.0.1 (https://cge.food.dtu.dk/services/PlasmidFinder/, (accessed on 24 June 2025)) [44], under specific settings (database Gram-positive; threshold for minimum 95% identity; minimum 60% coverage).

For predicting the mobilome, possible mobilome interactions and bacteria mobilome ARG relationships, the bioinformatic tool (with a metagenome assembly setting) at the following web address was used (https://tool2-mml.sjtu.edu.cn/VRprofile/home.php, version 2.0 (accessed on 24 June 2025)).

Virulence genes were analyzed by comparing them using the Virulence Factor Database (VFDB) (http://www.mgc.ac.cn/VFs/main.htm, version 4.0 (accessed on 24 June 2025)). Bacteriocin gene clusters and others clusters were predicted and visualized using antiSMASH bacterial, version 8.0.0 [45].

### 3.9. Statistical Analysis

In this study, descriptive statistics were performed in cases of antibiotic resistance, and all comparisons are presented numerically. For the antagonism activity of NCFSs against indicator bacteria and for biofilm formation, data were generated from independent experiments with at least eight replicates. Statistical significance was indicated using asterisks, with the following thresholds: *p* < 0.05 (*), *p* < 0.01 (**), and *p* < 0.001 (***). Microsoft Excel was used to collect and analyze the data. Data are presented in Tables as mean A (absorbance) ± SD (standard deviation) and were analyzed using t-tests.

## 4. Conclusions

*Ligilactobacillus salivarius* 48, isolated from the milk of a healthy dairy cow, satisfied safety requirements, including possessing no undesirable antibiotic resistance, enzymatic activity, and putative virulence factor genes. Additionally, *Ligilactobacillus salivarius* 48 exhibited essential probiotic properties, including inhibiting several pathogens, such as *Staphylococcus aureus, Escherichia coli* C 1971, *Salmonella enteritidis* CCM 4420, and *Bacillus cereus* CCM 869. WGS confirmed the presence of genetic information responsible for production of several bacteriocins, including a gene similar to salivaricin CRL1328 and one for β-galactosidase production. This gene could be involved in the transglycosylation of lactose, which results in the formation of highly desirable prebiotics known as galactose-oligosaccharides. In addition, this enzyme aids in the digestion of lactose. *Ligilactobacillus salivarius* 48 is proposed as a suitable candidate for protecting the mammary gland against not only *Staphylococcus aureus* but also other pathogens, including *Escherichia coli*, *Salmonella enteritidis*, and *Bacillus cereus.* Although additional investigations are required to evaluate the potential of the identified proteins, the findings presented in this paper may help in the design of promising new antibacterial strategies, either through the use of *Ligilactobacillus salivarius* 48 or its secreted proteins.

## Figures and Tables

**Table 1:** Distribution of the MIC, MIC_50_, and MIC_90_ values of eight antibiotics among studied lactobacilli strains (*n* = 40).

MIC (µg/mL)																
	0.03	0.06	0.12	0.25	0.5	1	2	4	8	16	32	64	128	256	MIC_50_	MIC_90_
Gentamicin					6	3	9	13	4	3					4	8
Kanamycin							1	1	3	7	7	9	6	4	64	128
Streptomycin								5	2	10	15	5	1		32	64
Neomycin						6	6	8	11	6	1				8	16
Tetracycline						3	15	16	2	1	1				2	4
Erythromycin		4	7	12	13	1		1							0.25	0.5
Clindamycin	3	3	11	8	3	8	2								0.125	0.25
Chloramphenicol			1				16	14	7						4	8

Legend: MIC—minimum inhibitory concentration; MIC_50_ and MIC_90_ were defined as the MIC that inhibited 50% and 90%, respectively, of the tested microorganisms.

**Table 2:** Enzymatic activity of the tested lactobacilli using API ZYM system.

Positive Enzymatic Activity (no)	Species (*n*)								
	*Ligilactobacillus salivarius* (15)	*Lactobacillus paracasei* (6)	*Ligilactobacillus agilis* (6)	*Lactobacillus casei* (3)	*Lactobacillus mucosae* (3)	*Lactobacillus ingluviei* (2)	*Lactobacillus fermentum* (2)	*Ligilactobacillus ruminis* (2)	*Lactobacillus plantarum* (1)
*alkaline phosphatase*				1					
*esterase*					2				
*leucine arylamidase*	10	6	2	3	2	2	1	2	1
*valine arylamidase*	2	6		3				1	
*cystine arylamidase*	2			1					
*acid phosphatase*	10	3	6	3		2			
*naphthol-AS-B1-phosphohydrolase*	9	4	2	1	1	1			1
*α-galactosidase*	5		1		2	2	2	1	
*β-galactosidase*	7		1	3	3	2	2	1	
*β-glucoronidase*				1					
*α-glucosidase*		6	1	1	2		1	1	
*β-glucosidase*	1		2	2	3			1	
*N-acetyl-β-glucosamidase*				1					

Legend: *n*—number of isolates belonging to an individual species; no—number of isolates positive to individual enzyme activity (only isolates with score 3–5).

**Table 3:** Antimicrobial activity of lactobacilli NCFSs.

NCFSs	A_600_ ± SD			
	*Staphylococcus aureus* (from Mastitis)	*Salmonella enteritidis* CCM 4420	*Escherichia coli* C 1971	*Bacillus cereus* CCM 869
**Control**	0.887 ± 0.008	0.806 ± 0.011	0.470 ± 0.063	1.043 ± 0.032
***Ligilactobacillus salivarius* 100/3**	0.139 ± 0.002 ***	0.125 ± 0.006 ***	0.150 ± 0.004 ***	0.125 ± 0.002 ***
***Ligilactobacillus salivarius* 48**	0.119 ± 0.002 ***	0.110 ± 0.006 ***	0.153 ± 0.005 ***	0.125 ± 0.004 ***
***Lactobacillus fermentum* 106**	0.133 ± 0.009 ***	0.096 ± 0.006 ***	0.159 ± 0.008 ***	0.117 ± 0.004 ***
***Lactobacillus paracasei* 53**	0.118 ± 0.005 ***	0.115 ± 0.013 ***	0.150 ± 0.002 ***	0.159 ± 0.012 ***
***Ligilactobacillus salivarius* 105/2**	0.118 ± 0.002 ***	0.120 ± 0.007 ***	0.149 ± 0.004 ***	0.129 ± 0.002 ***
***Lactobacillus paracasei* 1**	0.119 ± 0.002 ***	0.132 ± 0.007 ***	0.157 ± 0.002 ***	0.145 ± 0.002 ***
***Lactobacillus paracasei* 29**	0.385 ± 0.060 ***	0.353 ± 0.093 ***	0.157 ± 0.004 ***	0.735 ± 0.085 ***

Legends: neutralized cell free supernatants (NCFSs) from selected lactobacilli; A_600_—absorbance measurement at λ = 600 nm; For antagonism activity of NCFSs against indicator bacteria, data were generated from independent experiments with at eight replicates. Statistical significance was indicated using asterisks with the following thresholds: *p* < 0.001 (***). Microsoft Excel were used to collect and analyze the data. Data were expressed on a representative Table as mean (absorbance) ± SD (standard deviation) and statistically analyzed by *t*-tests.

## Data Availability

The datasets generated during and/or analyzed during the current study are available from the corresponding author on reasonable request.

## Supplementary Materials

The following supporting information can be downloaded at: https://www.mdpi.com/article/10.3390/ijms262110809/s1.

## Abbreviations

The following abbreviations are used in this manuscript:

BM	Bovine Mastitis
MALDI-ToF	Matrix-Assisted Laser Desorption Ionization–Time of Flight Mass Spectrometry
EFSA	European Food Safety Authority
FEEDAP	Panel on Additives and Products or Substances used in Animal Feed
MIC	Minimum inhibitory concentration
MIC_50_	Minimum inhibitory concentration that inhibited 50% of the tested microorganisms
MIC_90_	Minimum inhibitory concentration that inhibited 90% of the tested microorganisms
WGS	Whole- Genome Sequencing
CARD	The Comprehensive Antibiotic Resistance Database
CFSs	Cell–free supernatants
NCFSs	Neutralized cell free supernatants
CCM	The Czech Collection of Microorganisms
NCBI	National Centre for Biotechnology Information
BLAST	Basic Local Alignment Search Tool
CRISPR	Clustered Regularly Interspaced Short Palindromic Repeats
MGEs	Mobile genetic elements
RiPP	ribosomally synthesized and post-translationally modified peptides
ARGs	antimicrobial resistance genes
T3PKSs	bacterial type III polyketide synthases
VFDB	Virulence Factor Database
CFU	Colony Forming Units
PBS	Phosphate-Buffered Saline solution
MRS	De Man–Rogosa–Sharpe broth
MH	Muller Hinton broth
