# Supplementary material for: Whole-Genome Sequence Analysis and Subtractive Screening of Lactobacilli in the Searching for New Probiotics to Protect the Mammary Glands
Source: Int J Mol Sci. 2025 Nov 6;26(21):10809. doi: 10.3390/ijms262110809 (PMC12608424; doi:10.3390/ijms262110809)
Supplement: Supplementary file 1 [file ijms-26-10809-s001.zip › Table S6. ResFinder_genome ID_13_L. salivarius 48.docx]

Input Parameters

Input File 1: 13_o200.fa

Acquired antimicrobial resistance genes

Threshold for ID: 90.0 %

Minimum length: 60.0 %

Species and input data type

Selected species: Other

Database versions

ResFinder-2.5.1

Results

**ResFinder-4.7.2**

PhenotypesHide

other All

| **Antimicrobial** | **Class** | **WGS-predicted phenotype** | **Genetic background** |
| --- | --- | --- | --- |
| gentamicin | aminoglycoside | No resistance |  |
| tobramycin | aminoglycoside | No resistance |  |
| streptomycin | aminoglycoside | No resistance |  |
| amikacin | aminoglycoside | No resistance |  |
| isepamicin | aminoglycoside | No resistance |  |
| dibekacin | aminoglycoside | No resistance |  |
| kanamycin | aminoglycoside | No resistance |  |
| neomycin | aminoglycoside | No resistance |  |
| lividomycin | aminoglycoside | No resistance |  |
| paromomycin | aminoglycoside | No resistance |  |
| ribostamycin | aminoglycoside | No resistance |  |
| unknown aminoglycoside | aminoglycoside | No resistance |  |
| butiromycin | aminoglycoside | No resistance |  |
| butirosin | aminoglycoside | No resistance |  |
| hygromycin | aminoglycoside | No resistance |  |
| netilmicin | aminoglycoside | No resistance |  |
| apramycin | aminoglycoside | No resistance |  |
| sisomicin | aminoglycoside | No resistance |  |
| arbekacin | aminoglycoside | No resistance |  |
| kasugamycin | aminoglycoside | No resistance |  |
| astromicin | aminoglycoside | No resistance |  |
| fortimicin | aminoglycoside | No resistance |  |
| spectinomycin | aminocyclitol | No resistance |  |
| fluoroquinolone | quinolone | No resistance |  |
| ciprofloxacin | quinolone | No resistance |  |
| unknown quinolone | quinolone | No resistance |  |
| nalidixic acid | quinolone | No resistance |  |
| amoxicillin | beta-lactam | No resistance |  |
| amoxicillin+clavulanic acid | beta-lactam | No resistance |  |
| ampicillin | beta-lactam | No resistance |  |
| ampicillin+clavulanic acid | beta-lactam | No resistance |  |
| cefepime | beta-lactam | No resistance |  |
| cefixime | beta-lactam | No resistance |  |
| cefotaxime | beta-lactam | No resistance |  |
| cefoxitin | beta-lactam | No resistance |  |
| ceftazidime | beta-lactam | No resistance |  |
| ertapenem | beta-lactam | No resistance |  |
| imipenem | beta-lactam | No resistance |  |
| meropenem | beta-lactam | No resistance |  |
| piperacillin | beta-lactam | No resistance |  |
| piperacillin+tazobactam | beta-lactam | No resistance |  |
| unknown beta-lactam | beta-lactam | No resistance |  |
| aztreonam | beta-lactam | No resistance |  |
| cefotaxime+clavulanic acid | beta-lactam | No resistance |  |
| temocillin | beta-lactam | No resistance |  |
| ticarcillin | beta-lactam | No resistance |  |
| ceftazidime+avibactam | beta-lactam | No resistance |  |
| penicillin | beta-lactam | No resistance |  |
| ceftriaxone | beta-lactam | No resistance |  |
| ticarcillin+clavulanic acid | beta-lactam | No resistance |  |
| cephalothin | beta-lactam | No resistance |  |
| piperacillin+clavulanic acid | beta-lactam | No resistance |  |
| ceftiofur | under_development | No resistance |  |
| sulfamethoxazole | folate pathway antagonist | No resistance |  |
| trimethoprim | folate pathway antagonist | No resistance |  |
| fosfomycin | fosfomycin | No resistance |  |
| vancomycin | glycopeptide | No resistance |  |
| teicoplanin | glycopeptide | No resistance |  |
| bleomycin | glycopeptide | No resistance |  |
| lincomycin | lincosamide | No resistance |  |
| clindamycin | lincosamide | No resistance |  |
| dalfopristin | streptogramin a | No resistance |  |
| pristinamycin iia | streptogramin a | No resistance |  |
| virginiamycin m | streptogramin a | No resistance |  |
| quinupristin+dalfopristin | streptogramin a | No resistance |  |
| tiamulin | pleuromutilin | No resistance |  |
| carbomycin | macrolide | No resistance |  |
| erythromycin | macrolide | No resistance |  |
| azithromycin | macrolide | No resistance |  |
| oleandomycin | macrolide | No resistance |  |
| spiramycin | macrolide | No resistance |  |
| tylosin | macrolide | No resistance |  |
| telithromycin | macrolide | No resistance |  |
| tetracycline | tetracycline | No resistance |  |
| doxycycline | tetracycline | No resistance |  |
| minocycline | tetracycline | No resistance |  |
| tigecycline | tetracycline | No resistance |  |
| quinupristin | streptogramin b | No resistance |  |
| pristinamycin ia | streptogramin b | No resistance |  |
| virginiamycin s | streptogramin b | No resistance |  |
| linezolid | oxazolidinone | No resistance |  |
| chloramphenicol | amphenicol | No resistance |  |
| florfenicol | amphenicol | No resistance |  |
| colistin | polymyxin | No resistance |  |
| fusidic acid | steroid antibacterial | No resistance |  |
| mupirocin | pseudomonic acid | No resistance |  |
| rifampicin | rifamycin | No resistance |  |
| metronidazole | nitroimidazole | No resistance |  |
| narasin | ionophores | No resistance |  |
| salinomycin | ionophores | No resistance |  |
| maduramicin | ionophores | No resistance |  |

Acquired AMR gene hitsHide

| Resistance gene | Identity | Alignment length/gene length | Position in reference | Contig or depth | Position in contig | Phenotype | PMID | Accession no. | Notes |
| --- | --- | --- | --- | --- | --- | --- | --- | --- | --- |

Gene Alignments

Show gene alignments

Chromosomal mutations mediating AMRHide

Acquired disinfectant resistance gene hitsHide

| Resistance gene | Identity | Alignment length/gene length | Position in reference | Contig or depth | Position in contig | Phenotype | PMID | Accession no. | Notes |
| --- | --- | --- | --- | --- | --- | --- | --- | --- | --- |

Gene Alignments

Show gene alignments

No gene sequences found in resistant genes.

Downloads

Table downloads

Začátek formuláře

Download phenotypetable (txt)

Konec formuláře

Download acquired AMR gene results:

Začátek formuláře

Results as text

Konec formuláře

Začátek formuláře

Hit in genome sequences

Konec formuláře

Začátek formuláře

Resistance gene sequences

Konec formuláře

Začátek formuláře

Results as tabseperated file

Konec formuláře

Download Chromosomal point mutation results:

Začátek formuláře

Results as tabseperated file

Konec formuláře

Začátek formuláře

Results as text file
