# Supplementary material for: Whole-Genome Sequence Analysis and Subtractive Screening of Lactobacilli in the Searching for New Probiotics to Protect the Mammary Glands
Source: Int J Mol Sci. 2025 Nov 6;26(21):10809. doi: 10.3390/ijms262110809 (PMC12608424; doi:10.3390/ijms262110809)
Supplement: Supplementary file 1 [file ijms-26-10809-s001.zip › Table S1. Minimum inhibitory concentration values of the Lactobacillus sp. (1).docx]

| Isolates | Antibiotics (MICs - μg/mL) | | | | | | | |
| --- | --- | --- | --- | --- | --- | --- | --- | --- |
|  | Gm | Km | Sm | Nm | Tc | Em | Cl | Cm |
| *L. mucosae* 112/1 | 0.5 | 16 | 4 | 1 | **8 (R)** | 0.12 | 0.25 | 0.12 |
| *L. mucosae* 100/2 | 2 | 32 | 16 | 2 | 4 | 0.25 | 0.03 | 2 |
| *L. plantarum* 26 | 2 | 16 | 16 | 4 | 4 | **4(R)** | 0.12 | **8(R)** |
| *L. paracasei* 1 | 1 | 32 | 16 | 4 | 8 | 0.25 | 0.12 | 8 |
| *L. paracasei* 53 | 2 | 32 | 32 | 4 | 2 | 0.12 | 0.12 | 2 |
| *L. salivarius* 100/3 | 8 | 32 | 32 | 16 | 4 | 0.5 | 0.12 | 2 |
| *L. salivarius* 45 | 4 | **128(R)** | 32 | 4 | 4 | 0.25 | 0.12 | 8 |
| *L. fermentum* 106 | 2 | 2 | 16 | 2 | 4 | 0.25 | 0.06 | 2 |
| *L. agilis* 103/2 | 4 | **64(R)** | **64(R)** | 16 | 4 | 0.25 | 0.12 | **8(R)** |
| *L. ingluviei* 107/6 | 2 | **64(R)** | 32 | 2 | **32(R)** | 0.25 | 0.25 | **8(R)** |
| *L. agilis 101/2* | 4 | **256(R)** | 32 | 16 | 2 | 0.25 | 0.25 | **4(R)** |
| *L. casei 28* | 4 | **64(R)** | 32 | 8 | 2 | 0.12 | 2 | **4(R)** |
| *L. casei 74* | 4 | **64(R)** | 32 | 8 | 2 | 0.06 | 1 | **8(R)** |
| *L. agilis 101/2?* | **16(R)** | **256(R)** | **128(R)** | 32 | 4 | 0.25 | 0.25 | **4(R)** |
| *L. casei 79* | 4 | **64(R)** | 32 | 8 | 2 | 0.06 | 1 | **4(R)** |
| *L. paracasei 29* | 2 | 16 | 8 | 2 | 1 | 0.12 | 0.12 | 2 |
| *L. fermentum 16/4* | 1 | 16 | 8 | 1 | 2 | 0.12 | 0.03 | **4(R)** |
| *L. salivarius 105/2* | 2 | 32 | 16 | 4 | 2 | 0.12 | 0.06 | 2 |
| *L. paracasei 34* | 2 | 16 | 4 | 2 | 1 | 0.12 | 0.25 | 2 |
| *L. salivarius 55* | 4 | **64(R)** | **64(R)** | 8 | **16(R)** | 0.25 | 0.12 | 8 |
| *L. salivarius 89* | 8 | **128(R)** | 32 | 8 | 4 | 0.5 | 0.5 | **4(R)** |
| *L. salivarius 53* | 4 | **64(R)** | 32 | 4 | 2 | 0.5 | 1 | 2 |
| *L. salivarius 74* | 4 | **128(R)** | **64(R)** | 8 | 4 | 0.5 | 2 | 2 |
| *L. salivarius 51* | 8 | **256(R)** | 16 | 8 | 2 | 0.5 | 1 | 2 |
| *L. salivarius 96* | 4 | **128(R)** | **64(R)** | 16 | 4 | 0.5 | 1 | 2 |
| *L. salivarius 16/3* | 8 | **128(R)** | 32 | 8 | 4 | 0.25 | 0.25 | 2 |
| *L. salivarius 37* | 4 | **64(R)** | 32 | 4 | 4 | 0.5 | 1 | **4(R)** |
| *L. salivarius 17* | **16(R)** | **64(R)** | 16 | 16 | 4 | 0.5 | 0.5 | 2 |
| *L. salivarius 48* | 8 | 16 | 32 | 16 | 4 | 0.5 | 0.25 | 2 |
| *L. mucosae 114/3* | 0.5 | 8 | 4 | 1 | 2 | 0.5 | 0.12 | 2 |
| *L. agilis 239/3* | N | N | N | N | N | N | N | N |
| *L. plantarum 218* | N | N | N | N | N | N | N | N |
| *L. salivarius 237* | 4 | **256(R)** | **64(R)** | 8 | 2 | 0.5 | 1 | 2 |
| *L. ingluviei 240/1* | 2 | 32 | 32 | 8 | 4 | 0.5 | 0.5 | **4(R)** |
| *L. agilis 263* | 0.5 | 16 | 16 | 4 | 1 | 0.25 | 0.25 | **4(R)** |
| *L. paracasei 246/2* | 0.5 | 8 | 4 | 2 | 2 | 0.06 | 0.12 | **4(R)** |
| *L. ruminis 211* | 1 | 32 | **16(R)** | 1 | 2 | 0.5 | 0.06 | **4(R)** |
| *L. salivarius 236/2* | 4 | **128(R)** | 32 | 8 | 2 | 0.5 | 1 | **4(R)** |
| *L. paracasei 240/2* | 0.5 | 4 | 4 | 1 | 2 | 0.25 | 0.12 | 2 |
| *L. agilis 241* | 0.5 | 8 | 16 | 1 | 4 | 0.06 | 0.03 | **4(R)** |

Table S1. Minimum inhibitory concentration (MICs - μg/mL) values of the *Lactobacillus* spp.

Abbreviations: The minimum inhibitory concentration (MIC) of several antibiotics were determined using VetMIC (National Veterinary Institute of Sweden, Uppsala, Sweden) plates for LAB, containing serial 2-fold dilutions of 8 antibiotics Gm – gentamicin, Km – kanamycin, Sm – streptomycin, Nm – neomycin, Tc – tetracycline, Em – erythromycin, Cl – clindamycin, Cm – chloramphenicol, N – not detected. The bold and underlined values represented MIC value > than cut- off value; (R) resistant isolates
