# Supplementary figures and images for: Whole-Genome Sequence Analysis and Subtractive Screening of Lactobacilli in the Searching for New Probiotics to Protect the Mammary Glands
Source: Int J Mol Sci. 2025 Nov 6;26(21):10809. doi: 10.3390/ijms262110809 (PMC12608424; doi:10.3390/ijms262110809)

**Genome_13**


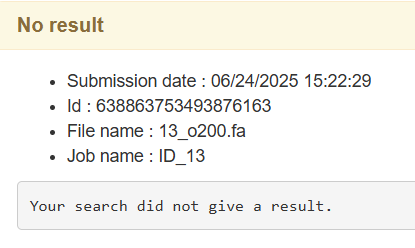

Supplement: Supplementary file 1 [file ijms-26-10809-s001.zip › Table S3. CRISPRCasFinder_results_L. salivarius 48.docx]

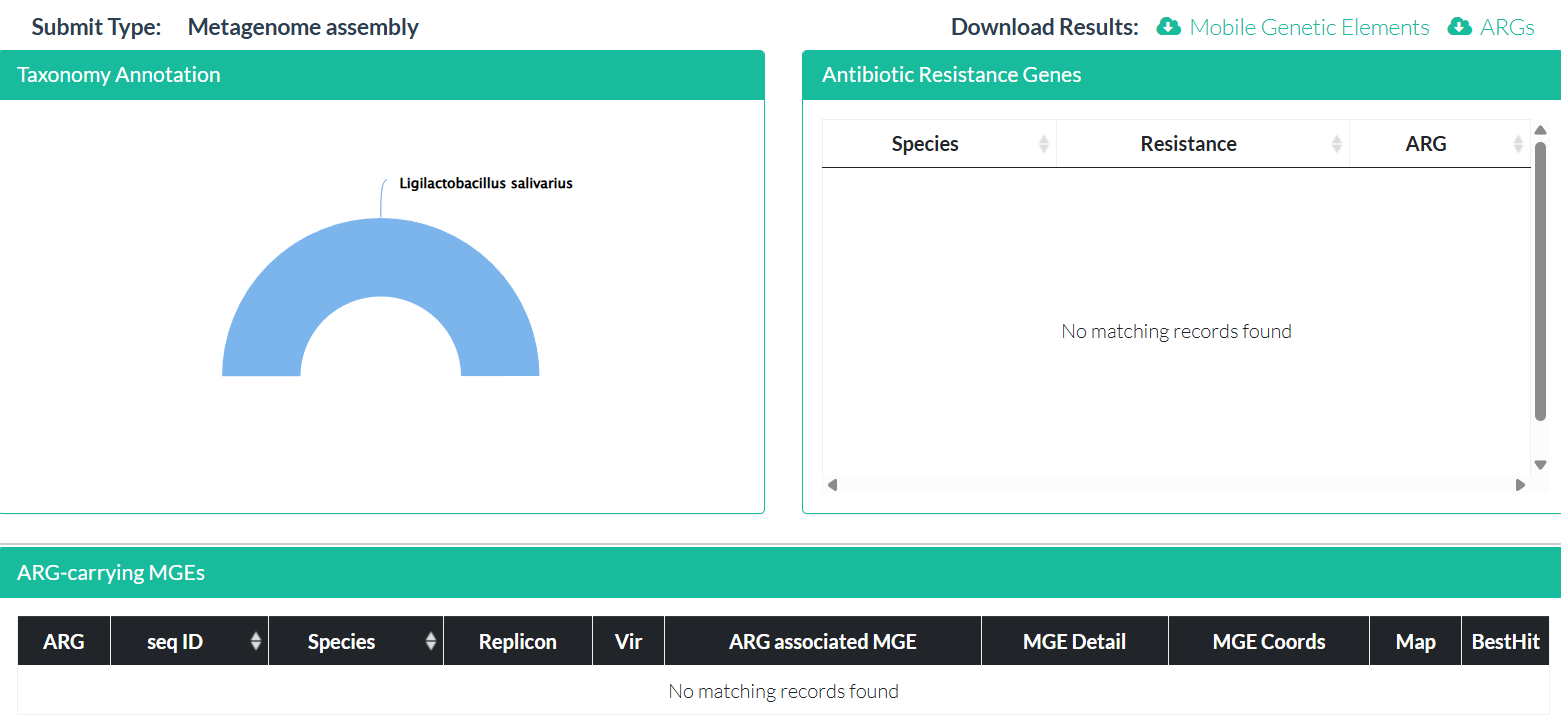

Supplement: Supplementary file 1 [file ijms-26-10809-s001.zip › Table S5. Mobilome Prediction_SampleID_13_L. salivarius 48.docx]
